# Charge Transfer Altered by Particle Deposition as a Contact Line Moves over a Hydrophobic Surface

**DOI:** 10.1021/acs.langmuir.5c05193

**Published:** 2026-02-18

**Authors:** Lars Egil Helseth

**Affiliations:** Department of Physics and Technology, University of Bergen, Allegaten 55, 5020 Bergen, Norway

## Abstract

An aqueous three-phase contact line moving over a hydrophobic surface is known to give rise to electrical charge transfer in a process that is sometimes referred to as slide electrification. Here it is shown that the charge transfer is significantly altered if the liquid contains small particles that adhere to the solid surface. For TiO_2_ nanoparticles in water, it is found that the charge transfer decays nearly exponentially to a very small value with a time constant that depends on the particle concentration. The increase in particle area coverage on the solid surface is correlated to the reduction in charge transfer, and a simple theory is developed to explain this behavior. Further studies of two different types of carbon particles in water reveal that the charge transfer initially increases before decaying even though the particle area coverage monotonously increases with time. It is suggested that this behavior is due to the release of ions, which increase the charge available to be transferred from the electrical double layer.

## Graphical abstract

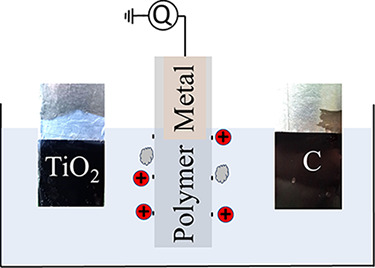

## Introduction

Contact electrification is known to occur when a hydrophobic polymer surface comes in contact with an aqueous solution.^1–10^ When the three-phase contact line moves across the solid surface, the initial surface wetting is governed by stick–slip motion that leads to irreversible increase in charge.^11^ Subsequent liquid motion and dewetting result in charge separation.^4,12,13^ Prewetted surfaces may also give rise to a repeatable charge transfer when exposed to oscillatory dipping motion, which can be explained by shear forces which separate charges from an electrical double layer at the solid–liquid interface.^14^ The charge transfer due to solid–liquid contact can be controlled by the surface roughness,^15^ its pre-existing charge state^16–22^ and the underlying substrate.^23^ However, also the liquid itself plays an important role, and it has been found that pH,^24,25^ ion concentration^24–29^ and presence of surfactants^30,31^ may alter the charge transfer significantly. Mixing water with other miscible liquids may alter the charge transfer in the presence of ions.^32^ Moreover, simultaneous processes may play a role. For example, it has been found that simultaneous dewetting of pairs of surfaces give rise to charges determined by both the expected dewetting charge separation as well as biased electrification.^33^ In general, it is of importance to understand the underlying physics in order to utilize solid–liquid contact electrification in devices,^34,35^ and reviews of recent applications in sensors and energy harvesting can be found in refs 36 and 37.

Electrostatic adhesion of micron-sized particles plays an important role in numerous industrial applications,^38^ and may also have significant impact on mechanisms for dry assembly of matter.^39,40^ In liquids, it is known that surfaces charged by contact electrification may give rise to particulate deposition through redox-reactions at the surface of polymers^41^ or reactant reduction by radicals on noble metals.^42^ Also, in absence of reactive species one has observed that dewetting leaves behind charge on the hydrophobic surface, which will cause particles to adhere such that the charge distribution can be visualized.^29,43,44^ Either charged toner powder,^29^ carbon particles^43^ or fluorophores^44^ have been used to visualize surface charge distributions after dewetting. In ref 44 it was demonstrated that positively charged fluorophores are more efficiently deposited at the hydrophobic surface than negatively charged fluorophores.

Despite these investigations, there is still a lack of knowledge of what happens to the charge transfer in the presence of particles adhered to the surface. Do the particles reduce charge transfer as more of the hydrophobic area is covered, or can it increase under certain circumstances? How fast is the charge transfer reduction, and how does the particle concentration influence the charge transfer? These and other questions are of interest in applications in which particles are present and may enhance or reduce the charge transfer significantly. Answering these questions will not only allow a better understanding of the underlying charge transfer mechanisms but also provide insight into how particles influence the performance of sensors and energy harvesting mechanisms utilizing solid–liquid charge transfer.

In the current work, the influence of particles on the charge transfer is investigated. Two different commonly available particles are selected, based on titanium dioxide and carbon, since they were tested and found to adhere to fluoropolymer surfaces in regions where the three-phase contact line had moved. The details of the adhesion mechanisms for the different particles are not under scrutiny in the current work but rather how the presence of particles alters the charge transfer observed due to a moving contact line, both dynamics and dependence on particle concentration in water.

## Materials and Methods

Deionized, ultrapure water (18.2 MΩcm, Millipore) was used to make all of the solutions. The particles dissolved in water were either TiO_2_ nanoparticles (Sigma-Aldrich, 21 nm particle size, 718467–100G), carbon nanopowder (Sigma-Aldrich, < 50 nm particles size, 633100–25G) or activated carbon particles (Sigma-Aldrich, activated charcoal SX2, particle sizes in the range 2–30 μm, 93067–250G). The carbon particles with <50 nm particle size will be referred to as C50, and the larger particles CNorit. Low particle concentrations (below 0.1 g/L) had very little influence on advancing, receding or static contact angles on hydrophobic fluorinated ethylene propylene (FEP), and were found to be in agreement with those reported in ref 15. At higher particle concentrations, there were some changes in contact angle, but a detailed investigation of this is outside the scope of this work. The pH of the solutions was monitored by using a pH meter (Hannah Combo, HI 98129). A Gamry ref 600 potentiostat was used to measure the impedance spectra and extract electrical parameters of the particle solutions, see the Supporting Information.

Charge transfer is measured using the dipping probe technique discussed in detail in refs 14, 27, and 45. In the current work, the probes are made by partially covering a 2 mm thick, 25 mm wide, and 50 mm tall black polystyrene substrate with 0.03 mm aluminum film. On top of this arrangement, a 50 μm thick FEP film (Dupont) was fixed using curable polydimethylsiloxane (PDMS). If the contact line is moved up and down near the position of the metal edge, this arrangement allows an increased change in charge and a better signal-to-noise ratio upon dipping as compared to a homogeneous system with an FEP-surface on top of an electrode that covers the entire polystyrene substrate.

The particle solutions were made by dissolving the particles in water and then using a pipettor to inject the needed concentration into the beaker in which the dipping probed was applied. Before use, the FEP-surface of the dipping probe was cleaned thoroughly with methanol and thereafter rinsed with water. An electromagnetic shaker (Smart Materials GmbH) was used to dip the probe into about 70 mL aqueous solution with an oscillation amplitude of approximately 1 cm at a frequency of 2.4 Hz, such that the velocity of the probe during dipping was about 0.1 m/s.^27,45^ An electrical wire was connected from the aluminum film of the probe to a Keithley 6514 electrometer, marked by a ‘Q’ in Figure 1a. As the probe moves either up or down, a current is flowing when the three-phase contact line passes the edge of the aluminum film, and the corresponding charge is measured by the electrometer.

**Figure 1:**
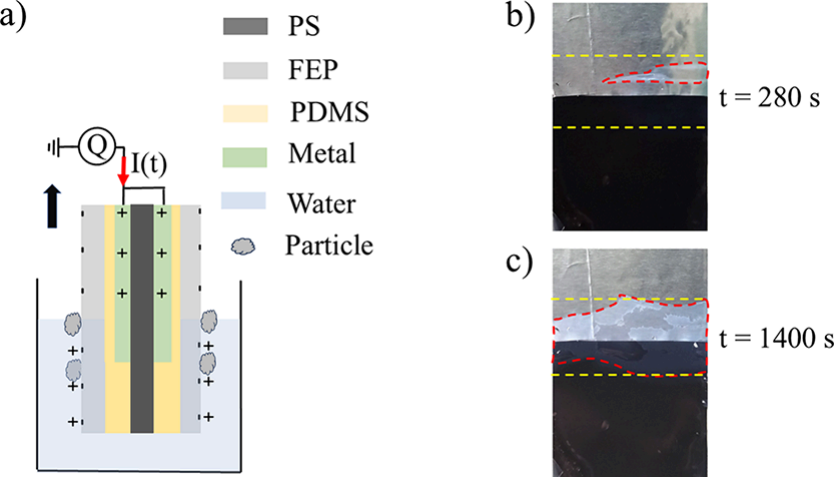
a) Schematic drawing of the experimental setup with the polymer-covered metal electrode dipped into water-based particle solution. In part b is shown an optical image of the surface of the dipping probe after 280 s moving up and down at a frequency of 2.4 Hz in 0.01 g/mL TiO_2_ particle solution. In c), the probe has moved up and down for 1400 s. The red dashed lines show the regions that have been covered by TiO_2_ particles, while the yellow lines indicate the range over which the contact line moves during periodic dipping. For reference; the black polystyrene (PS) substrate is 25 mm wide.

During dipping the probes in particle solutions, it was observed that the particles adhered to a hydrophobic solid surface, only in the region where the contact line had moved. An example is shown in Figure 1b, where a picture of the probe after dipping it in 0.01 g/mL TiO_2_ particle solution for 280 s at a frequency of 2.4 Hz. The probe was lifted out of the solution, and a picture was taken by a digital camera before lowering the probe back into the liquid and once more starting the dipping motion. The whole stop-start process took about 30–60 s. The contrast between the bare and particle-covered solid surface allowed one to extract the area covered by TiO_2_, as shown encircled in dashed red lines. In Figure 1c, the probe has moved up and down for 1400 s, thus giving rise to a significantly larger particle coverage.

When dipped in pure water, the probe used in the current work would generate a charge signal versus time, as shown in Figure 2a. The charge transfer, which is the difference between maximum and minimum measured charge, is about 4 nC for the probe used to obtain Figure 2a, and could remain like that to within approximately ± 0.2 nC for up to 2 h if left to operate in air. Evaporation and a CO_2_-dependent change in pH could cause a reduction in charge transfer below the uncertainty if the probe was left operating in air for longer periods, but all of the experiments reported here were finished before such effects could play a role.

**Figure 2:**
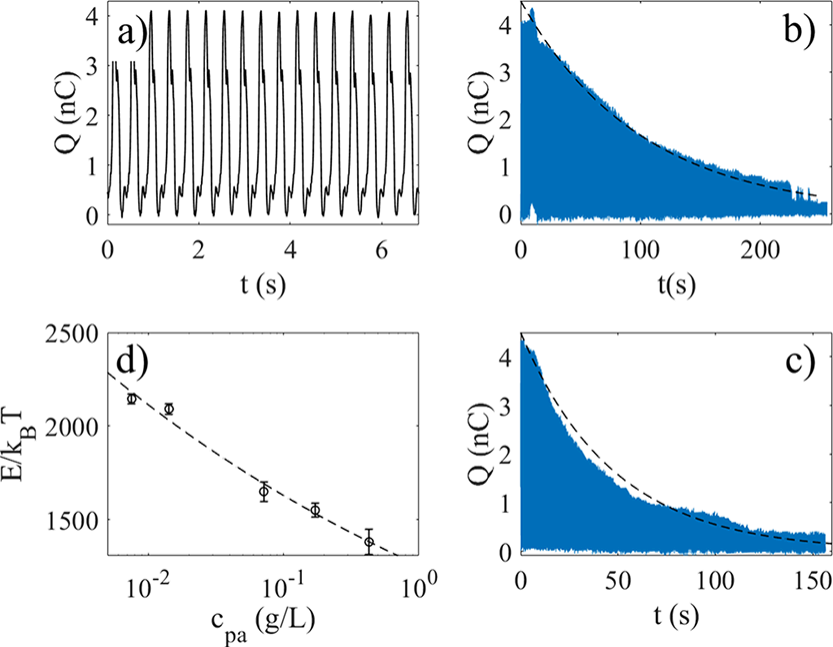
Charge measured as a function of time when the probe is dipped in pure water (a). If one injects 0.5 mL 10 g/L TiO_2_ solution (b) or 0.5 mL 60 g/L TiO_2_ solution (c) to 70 mL water, the charge decays as seen in b) and c), respectively. In d), the activation energy (relative to the thermal energy *k*_B_ *T*) is estimated using the time constants from experiments including those of b) and c), and the dashed line shows a fit of the function E/k_B_ *T* = E_0_c_pa_^α^, with E_0_ = 1.3·10^3^ and α=-0.11.

Upon adding aqueous particle solutions, particles would adhere to the FEP surface as shown in Figure 1b,c and the charge would noticeably and quickly change with time. An example wherein the charge is measured continuously during dipping (without taking pictures as in Figure 1) is shown in Figure 2b, where 0.5 mL of a solution of 10 g/L TiO_2_ particles was injected into 70 mL of deionized water, resulting in a nearly exponential decay of the charge as shown.

## Results and Discussion

### Charge Transfer in the Presence of TiO_2_ Particles

Titanium dioxide nanoparticles are usually considered hydrophilic and do not precipitate from water solutions at small concentrations. At low ionic strength the zero point of charge of TiO_2_ particles has been found to be about pH = 6, although there is some size-dependence,^46^ and moreover the particles may form aggregates when the conditions are altered.^47^ The low surface potential and surface susceptibility^48^ make them unlikely to adhere directly to the FEP surface under flow as used in the current work. However, the three-phase contact line gives rise to negative charging of the FEP surface, which attracts the TiO_2_ particles to be located in this region as seen in Figure 1c. The spatial charge distribution does have an impact on where and when the particles adhere, but this will not be studied here. Instead, we will attempt to correlate the fact that the particles adhere with the measured charge transfer.

When an aqueous solution of TiO_2_ particles is injected into water, the charge transfer is altered as shown in Figure 2b. Here, 0.5 mL of a solution of 10 g/L TiO_2_ particles was injected into 70 mL of deionized water at t = 0 s, after which a monotonous decay in charge transfer occurs. After 220 s the charge transfer start to fluctuate before falling below 0.3 nC.

In Figure 2c, 0.5 mL of a solution of 60 g/L of TiO_2_ particles was injected into 70 mL of deionized water at t = 0 s. In this case the decay was monotonously decaying until reaching a low value, with no obvious fluctuations. Similar monotonous decay was observed for many different concentrations, and they all appeared to correlate with the FEP surface being covered with TiO_2_ particles. At higher concentrations of particles (Figure 2c), it was less likely to observe fluctuations found at lower concentrations (Figure 2b). It could be that during steady decay there is a monotonous increase in particle coverage with time, whereas during fluctuations the particle coverage also fluctuates briefly.

Impedance spectroscopy as detailed in the Supporting Information can be used to estimate the particle concentration (in g/L) and ion concentration (in mol/L) with c = Gc_pa_, where G ≈ 10^–3^ mol/g is estimated for low TiO_2_ particle concentrations. For a concentration of 0.1 g/L this would imply an ion concentration 10^–4^ M, whereas 0.01 g/L is expected to give 10^–5^ M. It is known that adding TiO_2_ to water releases H^+^ ions.^46–48^ The pH was therefore measured for TiO_2_ solutions, and found to be pH = 6.1 for 0.014 g/L and pH = 5.2 for 0.14 g/L, the latter concentration being twice that used in Figure 2b. The decrease in pH could be due to H^+^ or other cations, since it is known that most cations reduce the pH to varying degree. In the experiments of Figure 2, it is also observed that it takes many cycles to reduce charge transfer, as opposed to the very few cycles observed when adding small amounts of diluted acid. In ref 27 it was found that for H^+^, which in that study was the only ion which solely reduced the charge transfer, the concentration had to be increased to above 10^–5^ M (i.e., pH < 5) for a significant reduction in charge transfer using a similar type of dipping probe as in the current study. Reducing the charge transfer to zero would require H^+^ ion concentrations about 10^–3^ M (i.e., pH ≈ 3).^27^ Since charge transfer reduction and particle adhesion are observed for particle concentrations below 0.01 g/L, it is unlikely that release of H^+^ ions plays any significant role in the charge transfer reduction observed. It is more likely that the particle adhesion covers up the available polymer area preventing charge transfer. The exact underlying mechanism which governs this blockage, and whether it is related to wetting properties of the particle layer, cannot be resolved by the measurements presented here and is therefore outside the scope of this work.

A simple theory to explain the experimental observations can be made by noting that initially the area that can be occupied by the particles is proportional to the total area A_0_ the three phase contact line moves over. After a certain time t, corresponding to N dips, an area A_c_ is covered by particles. The particles are assumed to block charge transfer associated with the FEP surface but may also facilitate charge transfer ΔQ_r_(A_c_) associated with the newly formed particle surface. Therefore, if the charge transfer correlates linearly with the area not covered by particles, one may write

$$\Delta Q\left(t\right)=Q_{0}\left(1-\frac{A_{c}}{A_{0}}\right)+\Delta Q_{r}\left(A_{c}\right)$$ 1

where Q_0_ is the initial charge at t = 0 s corresponding to a fully available area A_0_ of FEP over which the contact line is swept. Note that ΔQ is the charge transfer at any time and represents the difference between the maximum and minimum measured charge at that time in the Q-t graphs of Figure 2. The next step is to determine how the charge transfer changes with time. First, we notice that for all near-completely particle-covered FEP surfaces studied in this work, the charge transfer is small, comparable to the uncertainty level of the experimental setup, and it is therefore reasonable to set ΔQ_r_ (A_c_) = 0 as a first approach. Then, the probability of particle-adhesion to the fluoropolymer surface should be denoted as P_C_. For each dip, some of the surface is covered by particles such that the charge transfer ΔQ becomes smaller if the TiO_2_ particles are assumed to cover the FEP surface, rendering it unable to facilitate slide electrification. Since each experiment constitutes a large number of dips, a continuous approach is used wherein the rate dΔQ/dN represents the change in charge for a small number of dips dN. It is further assumed that this rate is proportional to a probability P_C_ and the charge transfer ΔQ itself, i.e. dΔQ/dN = -P_C_ΔQ. Since N = ft, where f is the dipping frequency (here f = 2.4 Hz) and t is the time elapsed, the measured charge transfer can be written as

$$\Delta Q\left(t\right)=Q_{0}e^{-t/\tau },\tau =\frac{1}{fP_{C}}$$ 2

It is seen that inserting eq 1 into eq 2 gives rise to A_c_=A_0_[1-exp­(-t/τ)]. The dashed line of Figure 2b shows a fit of eq 2 to the experimental data with τ = 100 s, whereas in Figure 2c it is τ = 48 s. The time constant τ is found to decrease with particle concentration. The author is not aware of any experiments wherein such a particle adhesion time constant has been measured, but for the positively charged surfactant CTAB it was demonstrated in ref 30 that the exponential decay time was of the order of one second. For particles, the time-scale is much longer. Most of the particle adhesion occurs in a well-mixed particle solution since mixing to a near homogeneous solution only requires a few seconds.

It should be noted that the theory leading to eq 2 assumes first-order kinetics of the particle coverage. However, from Figure 1b, it is observed that the particles start depositing in the vicinity of a nucleation site and that signs of island growth occur. It is also seen in Figure 2b,c that the charge transfer fluctuates such that at certain time intervals the charge drops significantly faster than exponential, while in other time intervals the charge is nearly constant. One could imagine that such an onset of sudden drops in charge transfer is associated with fast island growth, whereas slow changes occur when the island growth saturates in some way. The latter phenomenon appears to be in line with what is observed experimentally, i.e., that when the particle coverage remains nearly constant, the charge transfer does not change either. However, the dipping probe setup used in the current work does not allow tracking fast changes in particle area, and it is therefore not possible to state whether there is a correlation between fast island growth and a fast increase in charge transfer. It should also be pointed out that the experiments revealed that there may not only be a single island but also that several islands may appear simultaneously. The reason for these nucleation sites is not entirely clear, but they could be due to small scratches that trap particles, bumps that collect small droplets, or chemical inhomogeneities on the FEP surface. It was also observed that the islands could appear at random positions and not necessarily the same location each time. From Figure 2b,c it appears that the charge transfer decay is nearly exponential which one would expect from random particle deposition, which suggests that sudden onsets or slow saturation due to island growth is not dominating the kinetics. The model leading to eq 2 is therefore a reasonable starting point, and building more complex models is outside the scope of this work.

If the adhesion probability is assumed to follow to a Eyring or Kramers type of activation,^49,50^ one may write P_C_=exp­(-E/*k*_B_ *T*), where E is the activation energy barrier, k_B_ is Boltzmann’s constant and T is the temperature. The activation barrier can then be extracted using

$$E=k_{B}\mathrm{Tln}\left(\mathrm{f\tau}\right)$$ 3

Figure 2d shows the calculated activation barrier, using data for τ such as those in Figure 2b,c, for different particle concentrations (c_pa_). The dashed line is a fit of E/*k*_B_ *T* = E_0_c_pa_^α^, with E_0_ = 1.3·10^3^ *k*_B_ *T* and α=-0.11. The small value of α suggests a very weak concentration dependence of E, although it is not clear at this point why that is. It could also be that the gradual buildup of particles during dipping makes it easier for new particles to attach to the solid surface. However, it should also be pointed out that the assumptions leading to eq 3 are at most approximate since they rely on near-equilibrium systems and do not take into account fluid flow and strong particle interactions.

If the particles are not strongly enough attached to the solid surface, they will be removed by the hydrodynamic drag during dipping or by a receding contact line. The fluid of viscosity η passes the attached particles with a velocity v≈0.1 m/s and must do a work FΔ*x* to remove a particle from the surface, where F is the viscous force and Δx≈R is the size of the particle. Assuming Stokes law, F≈6πηRv, a rough estimate of the work done can be found using W ≈ 6πηR^2^v ≈ 2 ·10^2^ *k*_B_ *T*, with η ≈ 1 mPas and R ≈ 20 nm. The estimate for W, which is entirely due to drag by laminar fluid flow, is nearly an order of magnitude smaller than the activation energies estimated in Figure 2d. However, it should be emphasized that the simple estimate does not account for fluid mechanical interactions due to the vicinity of the fluoropolymer surface or the presence of other particles. The force exerted on the particles by the three-phase contact line scales with the surface tension γ and the particle dimension R, and an estimate of the force F = Rγ results in the work W ≈ γR^2^ ≈ 7 ·10^3^ *k*_B_ *T* with γ=0.072 N/m. The estimate of the work by the contact line is much larger than the fluid drag contribution and also 3–4 times larger than the values of Figure 2d. It should be emphasized that simple scale estimate of the work does not account for the contact angle, any details of their geometry or the possibility that they are clustered. It could also be that the assumption of P_C_=exp­(-E/*k*_B_ *T*) leading to eq 3 does not account for the nonequilibrium contact line dynamics and provides a too low estimate of the activation energy E. Further studies of the activation energy required could possibly be probed by a large range of dipping velocities, but this is outside the ability of the experimental setup used and also outside the scope of the current work.

In order to obtain further insight into the charge transfer process, start–stop measurements where performed. These experiments were performed like before by adding particle solutions to pure deionized water. The charge transfer was measured up to a certain time, after which the dipping probe had been dipped for N time, before the probe was stopped from moving and raised out of the liquid. A picture was taken of the probe using a digital camera (see Figure 1b,c), before lowering it into liquid for further dipping. This process was repeated 5–6 times until the charge transfer was almost zero. It took between 30 and 60 s for the probe to be raised out of the liquid and taken picture of.

The data of Figure 3a show measurements of the normalized charge transfer (ΔQ/Q_0_) after a certain number of dips, as described above, after injecting two different volumes of 10 g/L of TiO_2_ particles into 70 mL of deionized water at t = 0 s. The blue squares correspond to an experiment with injection of 0.1 mL of the TiO_2_ solution, whereas the black circles correspond to injection of 1 mL. The dashed lines show a fit of eq 2, with τ=480 and τ=47 s, correspondingly. As in Figure 2b,c, it is seen that the exponential decay of eq 2 provides a reasonable approximation to the experimental data. The time constants obtained using stop-start experiments as in Figure 3a exhibit similar concentration-dependence as in continuous experiments such as those of Figure 2b,c. By stopping the dipping, it could be that one perturbs the adhesion mechanisms significantly to give systematic deviations. Therefore, some control experiments were undertaken to check whether this was the case. For example, it was found that injecting 0.1 mL of 10 g/L of TiO_2_ particles into 70 mL of deionized water would give rise to τ=500 s if done continuously, whereas τ=480 s with stop-start as reported above. In both types of experiments, it was found that upon repeating experiments with the same concentrations could give rise to uncertainty in charge transfer of the order of 10%, and it therefore appears that any systematic deviations occurring as a result of stop-start experiments are masked by other factors that control the uncertainty in using the dipping technique to probe charge transfer.

**Figure 3:**
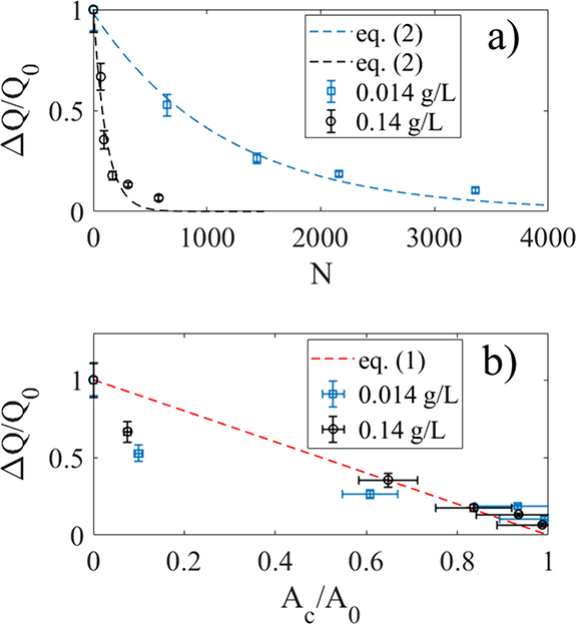
a) Measured normalized charge transfer (ΔQ/Q_0_) after N dips after injecting either 1 mL (black circles) or 0.1 mL (blue squares) of 10 g/L of TiO_2_ particles into 70 mL deionized water at *t* = 0 s. The legends show the corresponding homogeneous particle concentrations. The dashed lines are fits of eq 2 to the experimental data. b) The measured normalized charge transfer as a function of normalized particle-covered area (A_c_/A_0_) after 1 mL (black circles) and 0.1 mL (blue squares) of 10 g/L of TiO_2_ particles was injected into 70 mL deionized water. The dashed line represents eq 1.

The digital images taken during the start–stop measurements described above could be used to extract the area of the hydrophobic surface covered by particles. Examples of two such images are shown in Figure 1b,c, where the regions covered by particles are encircled in red. Here, A_0_ was selected as the total area over which the liquid slid as the dip probe moved up and down. This area was determined to be about 44% of that of the black polystyrene not covered by aluminum, seen in Figure 1b,c. The normalized charge transfer versus area is shown in Figure 3b for the same two experiments as reported in Figure 3a. The dashed line represents eq 1 with ΔQ_r_ = 0. There appears to be a correlation between charge transfer and particle area coverage, in reasonable agreement with eq 1. For small ratios A_c_/A_0_ there are systematic deviations that cannot be properly explained, although it could be due to a nonlinear deviation from eq 1. It could, for example, be that for A_c_/A_0_, particles already attached to the solid surface screen hydrodynamic flow and charge transfer, thus leading to a smaller charge transfer than expected from eq 1. It could also be that the bulk pH measurements made here do not accurately represent release of hydrogen ions when few particles have attached to the solid surface, and that in fact more H^+^ is released near the FEP surface during dipping resulting in a lower charge transfer than predicted by eq 1. In this study, no method was available to rule out such effects, and their determination is therefore outside the scope of this work.

It should be emphasized that extracting the particle-covered area relies on good optical contrast in the images. This can be challenging since the underlying aluminum electrode is not everywhere perfectly flat, giving rise to varying light reflection. Moreover, the hydrophobic surface is sometimes covered by a spatially inhomogeneous particle film, thus giving rise to a spatially inhomogeneous contrast. An example of the latter is shown in Figure 1c. While these contrast and measurements issues increase the uncertainty, it is unlikely that they can explain the deviation between theory and experiment in Figure 3b. It should also be mentioned that the nucleation growth mechanism seen in Figure 1a and observed for all types of particles causes the measured area A_c_ after a given time interval to vary between experiments with same concentration of particles. The reason for this is the more or less random occurrence of an island. After the probe is dipped many times, thus resulting in larger particle coverage exceeding 50% of the available area, the variation between experiments drops to typically less than 10%. For larger particle coverage, the formation of new islands may play a smaller role, thus allowing for better repeatability.

### Charge Transfer in the Presence of Carbon Particles

Carbon particles are chosen as the second type of particle used here. Activated carbon is an excellent adsorbent, and has been used extensively to remove dyes and purify water.^51^ The surface chemistry of the carbon particles plays an important role for the ions released into water,^52,53^ as does additional residual chemicals that remain in the powder supplied by the manufacturer.

Dipping the probe in a solution of C50 resulted in particle adhesion. This is shown in Figure 4a,b for two different probes, where in both cases 1 mL of 10 g/L C50 was added to 70 mL of DI water at t = 0 s.

**Figure 4:**
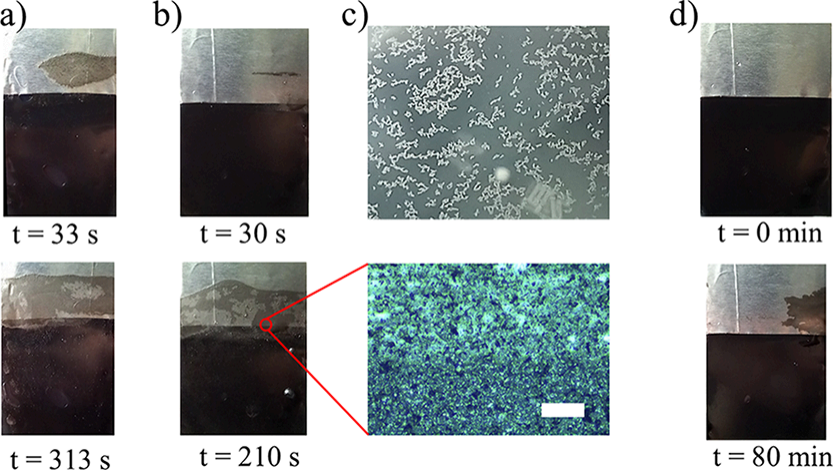
In a) and b), 1 mL of 10 g/L C50 was added to 70 mL of DI water, and the probe was dipped at a frequency of *f* = 2.4 Hz before digital pictures were taken at the times indicated. In c), the upper image shows the aggregation of C50 at the air–water interface, whereas the lower picture shows the C50 particles adhered to the FEP surface as viewed through an optical microscope. The scalebar is 100 μm. In d), 1 mL 150 g/L CNorit was added to 70 mL DI water and then the probe was dipped at a frequency of *f* = 2.4 Hz before digital pictures were taken at the times indicated.

In the two upper pictures in Figure 4a,b, taken at t = 33 s and t= 30 s, respectively, it is seen that in the first case the particle coverage is significantly larger than in the second case, implying faster adhesion in Figure 4a than in Figure 4b. From Figure 4a,b, it can also be seen that the regions covered by C50 particles are different for the two experiments. That is, the initial formation time of nuclei on which the particles can grow from and eventually cover the entire FEP surface appears to be varying from probe to probe. This could be related to surface inhomogeneities or different manners in which the probes enter the water, since it is impossible to make the probes entirely identical. However, the total covered area is similar in the two cases. In all experiments, such variations occurred early in the dipping process not only when changing probes, but also when repeating experiments with the same probe and the same solutions. However, eventually the surface was covered by particles in the region where the contact line receded, and the total time interval for this to happen was approximately the same as long as the particle concentration remained unchanged.

Comparing the pictures at t = 313 s and t = 210 s in Figure 4a,b, it is seen that the contrast is varying within the region covered by particles. Note that the regions in that appear to have ‘holes’ in the carbon layer, but are in fact covered by carbon. The appearance of ‘holes’ in the carbon film is caused by a combination of a spatially inhomogeneous carbon-film thickness and the reflection of light from water, as could more easily be recognized when inspecting the probes visually from different angles during taking the pictures or upon removing the film from water and letting it dry entirely.

Previous research has often regarded carbon surfaces as hydrophobic,^54^ although the underlying reason is debated and could be caused by contaminants.^55^ In the current work, it is observed that C50 particles not only exist in the bulk water, but also assemble clusters on the surface of the water, a feature not observed for TiO_2_ or CNorit. The clustering of C50 particles on the air–water interface is displayed in the upper picture of Figure 4c, wherein clusters on the order of 0.1 mm can be observed. As the three-phase contact line recedes over the FEP surface, the C50 forms particle aggregates on the solid surface as shown in Figure 4c. This image zooms in on a region of the dipping probe where the metal electrode (brighter region) ends and the black polystyrene (darker region) is in direct contact with FEP.

Figure 5a,b shows the normalized charge transfer ΔQ/Q_0_ as a function of N and A_c_/A_0_, respectively, after 1 mL of 10 g/L C50 was added to 70 mL of DI water in two different experiments corresponding to those displayed in Figure 4a,b. It is seen that the charge transfer increases for small N, and then decreases as N gets larger. This behavior cannot be explained by eq 2. Plotting the normalized charge transfer versus measured particle-covered area also showcases an initial increase, followed by a subsequent decrease. The experimental data show systematic deviations from eq 1, where the latter is displayed as a dashed line in Figure 5 b).

**Figure 5:**
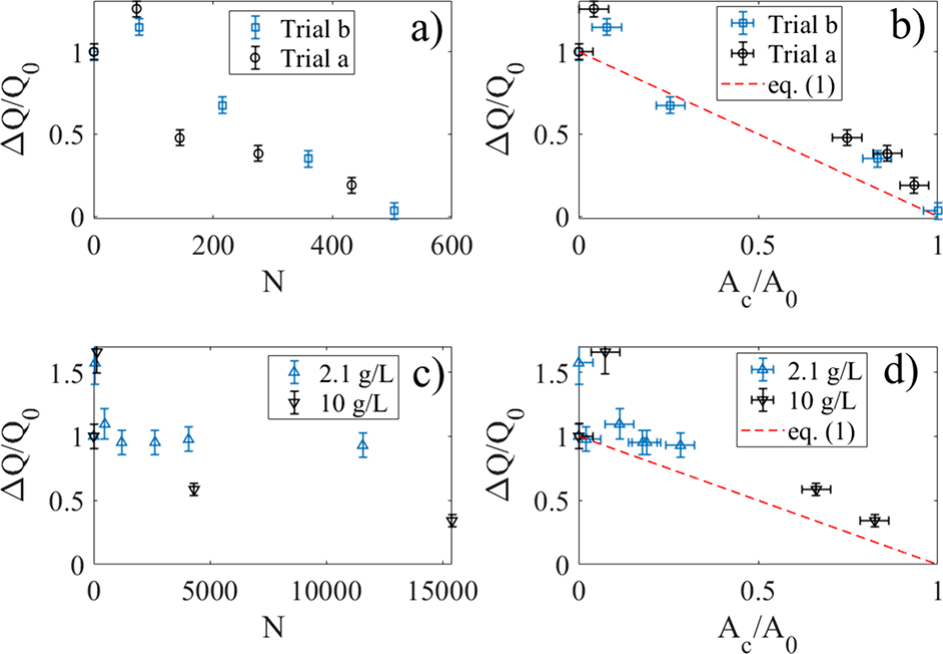
Normalized charge transfer as a function of N (a) and A_c_/A_0_ (b) after 1 mL of 10 g/L C50 was added to 70 mL of DI water. The blue squares and black circles correspond to the two different experiments of Figure 4a,b, respectively. In (c,d), the normalized charge transfer is displayed as a function N (c) and A_c_/A_0_ (d) after adding 1 mL 150 g/L CNorit (blue upward triangles) or 5 mL 150 g/L CNorit (black downward triangles) to 70 mL of DI water. The legends state the corresponding homogeneous particle concentrations. The dashed lines in b) and d) represent eq 1.

Figure 5c shows the normalized charge transfer for varying N (c) and A_c_/A_0_ (d) after adding 1 mL 150 g/L CNorit (blue upward triangles) or 5 mL 150 g/L CNorit (black downward triangles) to 70 mL DI water. We note two prominent features. First, it is found that in both cases in Figure 5c,d, the charge transfer increases with either N or A_c_/A_0_ initially, before any decay sets in, just as was seen also for C50 in Figure 5a,b. Second, the charge transfer does not become very small even after waiting for 100 min. One could imagine increasing the particle concentration even further, but under such conditions, there is obvious particle precipitation during the experiment, thus causing additional problems with accuracy of the measured concentration. Moreover, too high concentrations also caused an increased viscosity which could alter the dipping motion and cause additional problems, as discussed in ref 32. At the lower concentration in Figure 5c,d (blue upward triangles), the normalized charge transfer hardly decreased below 1 even after waiting for 80 min. The corresponding area covered by carbon is shown in Figure 4d and can be seen to cover only a small area. It is believed that this behavior is due to the relatively large particles and aggregates form when CNorit is mixed with water. These aggregates, while immersed in water, experience larger hydrodynamic drag and do not find sufficient options to adhere to the FEP surface due to size, geometry and surface chemistry, and do therefore not alter the charge transfer significantly.

Whenever TiO_2_ particle solution was added to water, one would observe a decrease in charge transfer as detailed in Figure 2b,c. However, upon adding either C50 or CNorit solutions to water, the charge transfer would always increase first and then start to decrease, as seen in Figure 5. A closer look at the charge transfer after adding C50 is displayed in Figure 6a, where 1 mL of 10 g/L C50 is added to 70 mL of water at t = 0 s, resulting in an increase in charge transfer from about 4 to 5 nC within a few seconds. Another example is shown in Figure 6b, where 5 mL of 150 g/L CNorit is added to 70 mL of water at t = 0 s, after which the charge transfer increases from 4 to approximately 7 nC in a few seconds. This increase in charge transfer is puzzling, since the area covered by carbon nanoparticles increases as well and is expected to reduce the charge transfer as in the case of TiO_2_.

**Figure 6:**
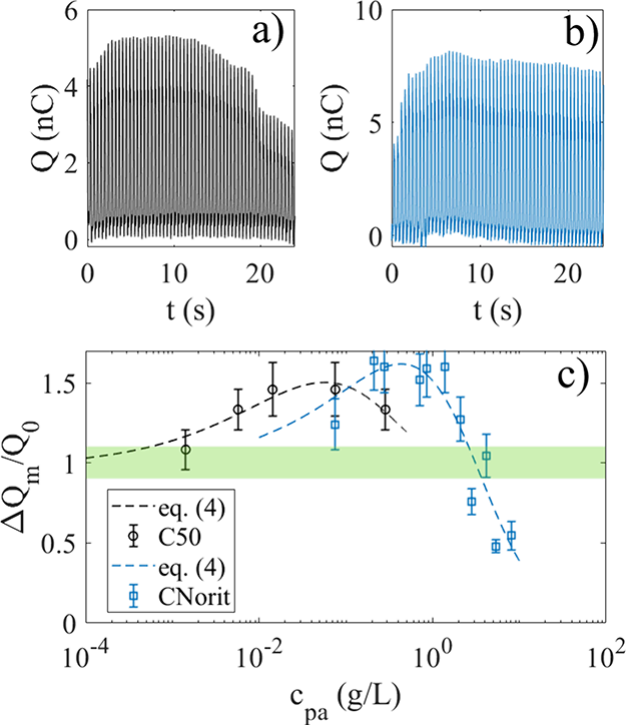
In a) the charge is recorded as 1 mL of 10 g/L C50 is added to 70 mL of water at *t* = 0 s, whereas in b) 5 mL of 150 g/L CNorit is added to 70 mL of water at *t* = 0 s. In part c, the normalized maximum charge transfer ΔQ_m_/Q_0_ (maximum difference between max and min values of charge) is recorded as a function of particle concentration c_pa_ for C50 (black circles) and CNorit (blue squares). The dashed lines are fits of eq 4 to the experimental data. The green rectangle shows the variability in the charge transfer measured in pure deionized water.

A possible explanation of the increase in charge transfer for carbon particles is an increase contact angle after the carbon particle solution is injected, thus causing an increase in charge transfer as predicted by the theory of ref 13. However, we also note that the carbon particles initially adhere to the FEP surface in islands, as seen in Figure 4. In regions not covered by particles, the contact angle does not change and this initially represent most of the FEP surface. Furthermore, Figure 5 shows that the relative increase in charge is much larger than the relative change in area, which suggests that an increase in contact angle cannot explain the data in Figure 6c.

An experiment to further investigate a possible origin for the initial increase in charge transfer reported in Figure 6b was undertaken by making a CNorit solution of 10 mL of 0.14 g/L in a test tube. The solution was left for 24 h to let all the particles sediment at the bottom of the test tube such that the fluid above the carbon particles was completely transparent. About 9 mL of the transparent supernatant was removed and then 9 mL new, deionized water was added to the test tube. This procedure was repeated three more times, and it was observed by impedance measurements that the electrical conductivity was reduced to about 10% of the initial value before washing. The charge transfer observed when dipping the FEP-covered probe in and out of an unwashed and washed solution of CNorit is shown in Figure 7a,b, respectively. It is observed that while for an unwashed solution the charge transfer increases by 25%, for a washed solution the increase is only 3%. This suggests that the initial increase in charge transfer is correlated with the ion concentration of the solution.

**Figure 7:**
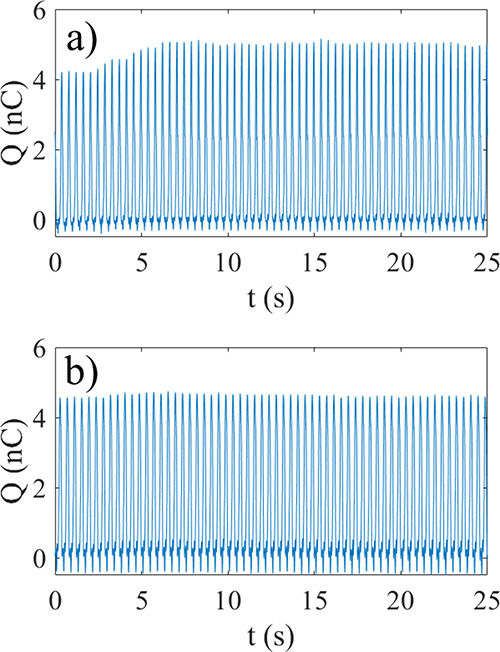
Initial change in charge transfer for unwashed (a) and washed (b) 0.14 g/L CNorit solutions added at *t* = 0.

A more likely explanation of the observed increase in charge transfer observed for carbon particles is, therefore, that free ions are also added with or induced by the carbon particles in Figures 5 and 6. It is known from ref 27 that adding small concentrations of positive metal ions or negative hydroxyl ions may increase the charge transfer. Upon injection of ionic solution, it takes a few cycles to reach maximum charge transfer, depending on the fluid dynamics distributing the ions throughout the liquid solution and, in particular, near the FEP surface. However, it should be emphasized that this process is much faster than particle adhesion kinetics, and the latter is believed to occur mostly in a homogeneous particle solution. Additional pH measurements demonstrated that adding 0.1 mL of 10 g/L C50 in 70 mL water resulted in pH = 6.4, 1 mL of the same solution in 70 mL water gave pH = 6.0, while 3 mL gave pH = 5.8. A reduction in pH also occurs when adding CNorit or TiO_2_, and it is therefore reasonable to state that added hydroxyl ions are not the reason for the increased charge transfer.

On the other hand, it is known that positive ions like Ca^2+^, Mg^2+^, K^+^ and Na^+^ often occur in solutions of acid-washed carbon particles,^52,56^ and these may be the reason for the behavior seen in Figures 5 and 6. To identify exactly which ions were released is not crucial since a range of different positive ions give rise to the same behavior as detailed in ref 27. However, it is possible to check whether the maximum charge transfer measured occur at similar ion concentrations as observed in ref 27.

The maximum charge transfer at different particle concentrations c_pa_ was recorded using measurements such as in Figure 6a,b. The results are displayed for CNorit (blue squares) and C50 (black circles) in Figure 6c, where Q_0_ is the charge transfer in pure water. It is noted from Figure 6c that the charge transfer due to C50 exhibits a peak in the range 0.01–0.1 g/L, after which it starts to decrease. It is also noted that only values of c_pa_ up to 0.3 g/L was recorded for C50, as higher concentrations gave a too fast changes in charge transfer (and area coverage) to reliable be able to detect a peak like in Figure 6a. On the other hand, CNorit did not give rise to a fast area coverage and rapid changes in charge transfer for any concentration, although here problems would occur with particle precipitation and viscous resistance toward the dipping probe at too high concentrations. For CNorit, the charge transfer peak in Figure 6c appears between 0.2 g/L and 1 g/L, i.e. considerably higher than for C50. The decrease in the maximum charge transfer above 1 g/L is significant, being only 50% of its original value at 10 g/L.

In the Supporting Information, impedance spectroscopy is used to relate the particle concentration (in g/L) and ion concentration (in mol/L) with c = Gc_pa_, where G ≈ 10^–3^ mol/g for C50 and G ≈ 5·10^–4^ mol/g for CNorit. This suggests that 1 mmol ions is released per gram of C50 particles, which would imply that 0.01 g/L gives rise to an ion concentration 10 μM. From Figure 6c it is seen that the maximum charge transfer for C50 occurs for c_pa_ around 0.01–0.1 g/L and for CNorit 0.2 g/L – 1 g/L, corresponding to ion concentrations in the range 10 −100 μM and 100–500 μM, respectively. This is in reasonable agreement with ref 27, where it was demonstrated that a peak in charge transfer was observed for a range of different ions at concentrations of about 100 μM – 1 mM.

An attempt to model the data in Figure 6c is made using the theory presented in ref 27 In this theory, the charge transfer is due to removal of ions of concentration c from the electrical double layer as the water–solid contact line passes over the hydrophobic surface. Ions in the electrical double layer located beyond a shear distance x_s_ are removed from the FEP surface thus introducing an additional charge transfer which adds to the value Q_0_ from pure water. At larger concentrations, quenching reduces water activity by a quenching factor γ_p_ = 1/(1+K_qp_c), where K_qp_ is the equilibrium quenching constant.^24^ In ref 27 the total charge transfer due to ions alone was written as

$$\Delta Q_{m}\left(c\right)\approx \frac{1}{1+K_{\mathrm{qp}}c}\left(Q_{0}-2.3\mathrm{AB}\sqrt{c}e^{-3.3x_{s}\sqrt{c}}\right)$$ 4

Here A is the area over which the contact line is sweeping and B a constant associated with the surface potential. Note that eq 4 does not account for the particle adhesion but only considers the influence of ions. The dashed black line of Figure 6c shows a fit of eq 4 to the experimental data for C50 with c = Gc_pa_, G ≈ 10^–3^ mol/g, Q_0_ = 4·10^–9^ C, x_s_ = 4·10^–8^ m and AB = −8 ·10^–8^ Vm^2^. The blue dashed line in Figure 6c is a fit of eq 4 to the experimental data (blue squares) for CNorit with c = Gc_pa_, G ≈ 5·10^–4^ mol/g, x_s_ = 9·10^–9^ m, AB = −4·10^–9^ Vm^2^ and K_qp_ = 772 M^–1^.

For a given mass concentration of particles, C50 gives rise to higher ionic conductivities than CNorit, and it could possibly be a result of the larger surface area provided by the former. It is noted that the quenching constant K_qp_ is comparable in magnitude to that reported previously for various ionic species, thus suggesting that the observed quenching at high concentrations follow that observed for a range of ions.^27^ The value of AB for both C50 and CNorit is approximately an order of magnitude lower than in ref 27. Note that AB is the product of the area (A) over which the charge is removed from the fluid and the constant B which in the Gouy–Chapman theory depends on the surface potential φ_d_ as $B\approx -\frac{4k_{B}T}{e}\tanh\left(\frac{e\varphi_{d}}{4k_{B}T}\right)$ ,^27,57^ where e is the electronic charge, k_B_ is Boltzmann's constant and T is the temperature. It is possible that the presence of particles may lower the surface potential. It is, however, also possible that they somehow alter the area A over which the charge is collected. It is not possible to distinguish these two effects within the simple theory of eq 4.

The shearing distance x_s_ = 4·10^–8^ m for C50 is comparable to values found in ref 27 for common ions such as Na^+^, K^+^, Cu^2+^ and so on. On the other hand, the value x_s_ = 9 nm extracted for CNorit is considerably smaller and in fact comparable in magnitude to the value found for H^+^ in ref 27. There is no clear mechanism suggesting such a small value of x_s_ for CNorit, since the particles are large and the ions presumably not H^+^ but rather larger ions that should give rise to a larger shear distance x_s_. Further investigations of different particle types with a very well-controlled release of ions might be helpful if future investigations are to provide better insight into the exact mechanism.

## Discussion of Control Parameters Determining the Charge Transfer and Particle Coverage

Upon use of the dipping probe setup in Figure 1, the range of motion of the three-phase contact line around the asymmetric metal electrode located under the FEP film determines the charge transfer as the probe is moving in and out of water. The maximum modulation is found when the contact line moves directly over the metal edge, as demonstrated experimentally and by modeling in ref 14. This fact has been used by the author to investigate charge transfer in solvents^32^ and aqueous solutions in the presence of salt^27^ or surfactants.^30^ However, it should be emphasized that this is just a technical choice and that particle deposition as reported in this work occurs independently of whether there is a metal edge or not below the hydrophobic surface. It is the range over which the contact line moves which determines the region of particle deposition, and an example of this is shown in Figure 8a, where the contact line moved in an area on the FEP surface away from the metal electrode edge. The experiment was the same as reported in Figure 2b, but with the probe immersed further into the solution. The initial charge transfer was about half of that in Figure 2b and decreased to a small fraction of the initial value in 250 s, in reasonable agreement with the kinetic observations of charge transfer in Figure 2b. In general, the position of the metal edge relative to the moving contact line was not found to influence the particle deposition significantly. In the current work the movement range of the contact line was kept relatively fixed and symmetrical about the metal edge in order to ensure that the charge modulation amplitude did not change significantly between experiments. Before every measurement, this range was adjusted visually, and the charge modulation in deionized water was monitored to ensure that the probe gave repeatable charge transfer measurements.

**Figure 8:**
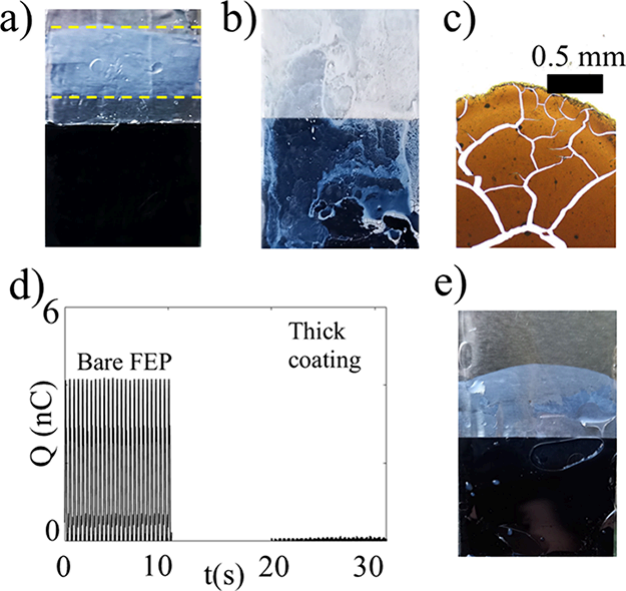
In a), 0.5 mL of 10 g/L TiO_2_ was injected in 70 mL water while the probe was moving up and down at a frequency of 2.4 Hz for about 250 s. The yellow lines indicate the range over which the contact line moves during periodic dipping. In b) a thick TiO_2_ coating was smeared on the FEP and left to dry on the FEP surface, while in c) a transmission microscopic image of the dried TiO_2_ pattern (the color is brown due to scattering) is shown. In d), the charge modulation with and without the thick coating in pure water is shown. In part e, 1 mL of 60 g/L TiO_2_ was injected in 70 mL of water while a probe covered with polydimethylsiloxane (PDMS) was moving up and down at a frequency of 2.4 Hz for 300 s.

It is known from the literature that the particle deposition depends on the orientation of the surface.^58^ In the current work, a vertical dipping probe is used. Upon injecting particle solution into water, the particles will in absence of any forced flow undergo sedimentation according to Stokes law giving the velocity v_s_=(ρ_p_-ρ_w_)­D^2^g/(18η), where ρ_p_ is the particle density (approximately 4·10^3^ kg/m^3^ for TiO_2_ and 2·10^3^ kg/m^3^ for carbon), ρ_w_ is the water density (approximately 1·10^3^ kg/m^3^), D is the particle diameter and η is the viscosity (about 10^–3^ Pas). For the particles used here, v_s_ ranges from 1 nm/s (single 21 nm TiO_2_ particles) to 1 mm/s (for the largest 30 μm CNorit particles). Note that the gravitational-induced sedimentation velocities are parallel to the FEP surface and much smaller than the typical velocities taken on by the probe pushing away water (0.1 m/s). While sedimentation of particles plays a minor role, it should be mentioned that gravitation allows for drainage of water on the FEP surface during contact line recession. However, the removal was not complete, and small droplets containing particles were also observed to remain on the surface when the sample was removed from water.

The experiments reported above suggest that the dipping probe used here only allows particle deposition upon contact line motion. If this is correct, then particles do not adhere on the vertical FEP surface at rest in the liquid. To test this, the probe was gently immersed in 0.07 g/L TiO_2_ solution for 45 min and placed at rest there, after which it was gently moved out of the solution and inspected. No particles could be observed by the eye or in a microscope, and the charge transfer was not altered upon redipping in deionized water. Similar observations were made for the carbon particles, which suggests that contact line motion is needed to deposit the particles. Although placing the probe in and removing it from the solution, thus allowing the contact line to recede once over the FEP surface, this is not enough to create notable particle adhesion.

If the surface is coated with particles before dipping is commenced, then very little charge transfer is observed. This was tested by placing the FEP surface on the probe horizontally and then dripping/smearing TiO_2_-saturated water solution across the surface, resulting in a thick (≈ 0.1 mm) coating as seen in Figure 8b. Observations in an optical transmission microscope reveal that the dried, thick coating consists of cracked layers of TiO_2_ as shown in Figure 8c. The TiO_2_ coating is very hydrophilic, and the contact angle is below 5^◦^ such that water spreads over the surface in a thin layer. Upon dipping the probe with the thick TiO_2_ coating in deionized water, the charge transfer is measured to be approximately 0.1 nC - 0.2 nC as seen in Figure 8d, comparable to that found for the dip-coated particle film in Figure 2b. The dipping probe measurement system used provides an uncertainty of about ± 0.2 nC after repeated measurements, which means that the charge transfer of a fully particle-covered FEP probe is barely in the range that can be measured with the available setup. The measurement of charge transfer on the thick TiO_2_ coating, combined with the measurements presented above, suggest that the charge transfer on hydrophilic particulate TiO_2_ surfaces is very small. The approach of setting ΔQ_r_ = 0 in eq 1 is therefore justified. The fact that charge transfer is small when the contact line is moving over hydrophilic surfaces is well-known in the literature, where a possible mechanism has been detailed in ref 13, and is therefore not investigated further here.

The results reported in the current study are all related to a single type of surface, namely, FEP. The reason for using this particular polymer is that it provides a large contact electrification, is stable over time, and is easily commercially available. Moreover, the author has investigated it repeatedly over time on different systems using the dipping probe technique.^27,30,32^ As reported in refs 29, 43, and 44, particle, and dye deposition is also possible on other types of surfaces. Yet another example is shown in Figure 8e, where a solution-deposited and cured (at room temperature) 0.1 mm thick film of polydimethylsiloxane (PDMS) was used as the outer film covering the metal electrodes instead of FEP. PDMS is also hydrophobic with a static contact angle of approximately 90^◦^, and particles adhere to such a probe when moving up and down in a solution. However, the charge modulation in water is rather small for PDMS (Q_0_ ≈ 0.3 nC) and in particle solution it falls quickly to less than the uncertainty before full particle coverage, thus rendering it hard to obtain reliable results. Improving the technique to achieve higher signal-to-noise ratio in order to test charge transfer versus particle adhesion on such surfaces was considered outside the scope of the current work.

In this study, the frequency was kept fixed at 2.4 Hz. This was done since it has been found that this frequency and a small range around it allows good signal-to-noise ratio and repeatable charge transfer measurements with the current experimental setup.^27,30,32^ Increasing the frequency typically results in more unwanted fluctuations in the cantilever and also splashing or drop formation, as the probe impacts the solution. On the other hand, the dipping probe setup allows measurements at slightly lower frequencies, down to approximately 1.2 Hz below which the amplitude of the contact line motion is so small that the charge modulation decreases (since the amplitude of the contact line motion of the cantilever cannot be maintained using the available equipment). Figure 9a shows the particle-covered FEP surface of the probe after injecting 0.5 mL of 10 g/L TiO_2_ solution at t = 0 s, dipping the probe at 1.2 Hz and waiting for 900 s. Figure 9b shows the corresponding charge versus time. It is seen that it takes about 900 s for the charge transfer to drop to about 0.2 nC. This should be compared to Figure 2b, where it takes only about 250 s to drop to 0.2 nC with a frequency of 2.4 Hz and otherwise similar conditions, which is less than one-third of the time-scale in Figure 9b. This is one example of a trend that appears when using lower frequencies; namely that it takes longer time to obtain significant reduction in charge transfer and nearly full particle coverage. The exponential does not fit the time-dependent charge in Figure 9b as well as for the results obtained at 2.4 Hz, which might be due to an initial nucleation barrier causing slower initial particle adhesion. Nonetheless, one could attempt to explain the time scale increase upon reducing the frequency by resorting to eq 2 and τ = 1/fP_C_. If the frequency is reduced by a factor of 2 (from 2.4 to 1.2 Hz), the time constant τ must increase by a factor of 2 if P_C_ remains constant and eq 2 is valid. This appears to not be the case, and one may speculate whether P_C_ also decreases with frequency due to weaker surface trapping caused by slower fluid flow such that time constant τ is larger than expected. The charge transfer and particle adhesion are likely to depend on the dipping frequency, which is the reason that the dipping frequency used in the current study was kept fixed.

**Figure 9:**
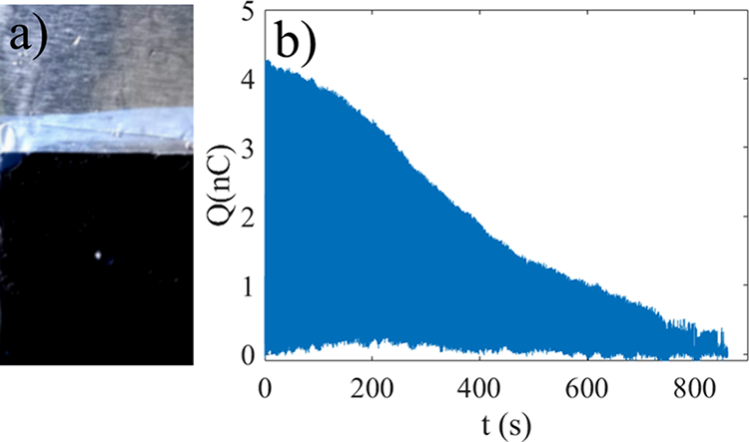
In a), 0.5 mL 10 g/L TiO_2_ solution was injected into 70 mL pure water and the probe oscillating at 1.2 Hz for 900 s before the picture was taken. In part b, the corresponding charge versus time is shown.

It is known that the charge transfer is strongly influenced by addition of acid,^24,27^ and further information on how acid influences a particle-free solution is reported in ref 27. In order to see how acidity influences the particle deposition, charge transfer and particle deposition was investigated for different concentrations of hydrochloric acid (HCl). The results are shown in Figure 10a. Here, 0.5 mL of 10 g/L TiO_2_ was injected in 70 mL of 0.7 mM HCl (upper image) or 54 mM (lower image) while the probe was moving up and down at a frequency of 2.4 Hz for about 290 s. In pure water, the particle coverage was almost complete, as reported in Figures 2 and 3. However, the images in Figure 10a demonstrate that the particle coverage decreased significantly as the acid concentration increased.

**Figure 10:**
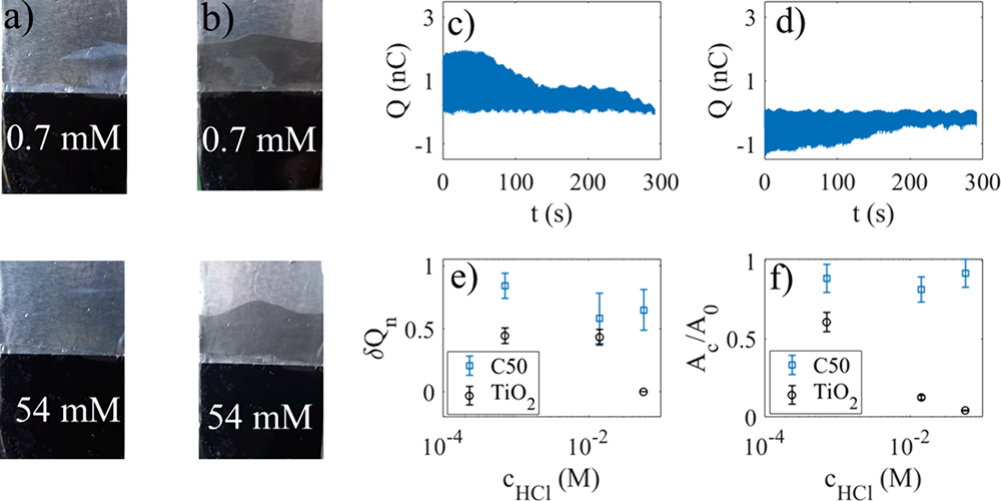
In a), 0.5 mL of 10 g/L TiO_2_ was injected in 70 mL 0.7 mM (upper image) or 54 mM HCl (lower image) while the probe was moving up and down at a frequency of 2.4 Hz for about 290 s before taking the picture. In part b, 1 mL of 10 g/L C50 was injected in 70 mL of 0.7 mM (upper image) or 54 mM HCl (lower image) while the probe was moving up and down at a frequency of 2.4 Hz for about 290 s before taking the picture. The corresponding charge modulation versus time is shown in parts c and d), respectively. In e) and f, the relative change in charge transfer over 290 s (e) and area coverage (f) versus acid concentration is shown for TiO_2_ particle (black circles) and C50 (blue squares).

In Figure 10b, 1 mL of 10 g/L C50 was injected in 70 mL of 0.7 mM (upper image) or 54 mM HCl (lower image). Here, the acid has no significant influence on the particle coverage. Figure 10c shows charge modulation in the presence of 0.7 mM HCl. It is observed that the charge transfer first increases just as reported in Figure 6a, after which it decreases gradually with certain jerks and a longer stalling (and slightly increasing) period between 150 and 250 s. These deviations from monotonous decay may be due to island growth. Figure 10d shows the charge transfer as C50 is injected into 54 mM HCl, and here the initial charge transfer has switched sign since one is below the isoelectric point, as was discussed in ref 27. Here, the release of ions when C50 is injected appears to result in a decreased absolute value of the charge transfer, which could possibly be due to the ions counteracting the effect of the H^+^ ions. However, we also note that in both Figure 10c,d the absolute value of the amplitude eventually decreases significantly with time which is probably due to an increase in carbon particle coverage with time, just as seen for pure water.

The relative change in charge transfer δQ_n_ is plotted for three different acid concentrations in Figure 10e for TiO_2_ particles (black circles) and C50 (blue squares). Here, δQ_n_ = (Q_0_-Q_290_)/Q_0_, where Q_0_ is the initial charge at the start of the particle injection and Q_290_ is the charge after 290 s. The particle coverage was also measured after about 290 s and is plotted versus acid concentration in Figure 10f. Here A_c_ is the area of particles extracted from pictures after removing the dipping probe from the solution after about 290 s, whereas A_0_ is the entire area swept by the contact line. It is seen that for TiO_2_ particles both δQ_n_ and A_c_ decrease with increasing acid concentration, thus suggesting that acid prevents deposition on the particles. This could be due to weaker negative or even positive FEP surface contact charge, preventing electrostatic forces to attract TiO_2_ particles to the FEP surface. Since TiO_2_ surfaces themselves have an isoelectric point at pH about 6,^46–48^ a positive charge at lower pH may weaken the attraction if the FEP surface acquires charge of the same sign. On the other hand, for C50 particles it is observed that particle deposition is not much influenced by acid concentration, and both δQ_n_ and A_c_ change much less than for TiO_2_ particles. This may suggest that the adhesion of carbon particles is much less sensitive to the electrostatic interactions tuned by pH changes. Carbon surfaces are often hydrophobic,^54^ caused by either active surface sites or hydrocarbon contaminants,^55^ and under such circumstances van der Waals may play a more prominent role. However, further speculations about the detailed interactions causing particle adhesion are outside the scope of the current work.

## Conclusion

In this work, it is demonstrated that particles adhered to a hydrophobic polymer may block the charge transfer contributed by the electrical double layer, as the aqueous contact line is moving past the solid surface. The particles must adhere sufficiently strongly to the solid surface to prevent them from being removed by the fluid motion. It is found that the energy barrier for adhesion decreases with the particle concentration, which can be explained by the interaction between the particles during adhesion. It is also demonstrated that the reduction in charge transfer correlates with the area that is occupied by the particles. If additional ions are released simultaneously with the particles, they may enhance the charge transfer.

The work presented here may have an impact on sensors and energy harvesting devices that utilize solid–liquid contact electrification. Particles are present in many different types of solutions and may adhere to the surface whether one wishes it or not, and it is therefore of interest to understand the consequences of such behavior. In the current study, there is evidence that particles themselves were only able to reduce charge transfer. However, in energy harvesting devices, one would like to increase charge transfer. Future work in this respect could aim at investigating particles with different wetting properties, for example, Janus particles, ion-releasing species, or particular structures that allow more efficient charge transfer.

## Supplementary Material



## Notes

Electrical impedance spectroscopy data (PDF)
