# Supplementary material for: Charge Transfer Altered by Particle Deposition as a Contact Line Moves over a Hydrophobic Surface
Source: Langmuir. 2026 Feb 18;42(8):6070–80. doi: 10.1021/acs.langmuir.5c05193 (PMC12961930; doi:10.1021/acs.langmuir.5c05193)
Supplement: Supplementary file 1 [file la5c05193_si_001.pdf]

Supporting Information

Charge transfer altered by particle deposition as a contact line moves over a hydrophobic surface

Lars Egil Helseth

Department of Physics and Technology, Allegaten 55, 5020 Bergen, University of Bergen, Norway

Number of pages: 7

Number of figures: 3

Number of tables: 1

Electrical impedance measurements

A Gamry Ref 600 in potentiostatic mode with zero bias and sinusoidal excitations of amplitude 150 mV was used to obtain the impedance spectra. Electrical impedance spectra were measured in a cuvette in which indium tin-oxide (ITO) electrodes were glued to the walls using polydimethylsiloxane (PDMS) as shown in fig. S1 a). About 0.8 mL of particle solutions was filled in the cuvette. The distance between the ITO electrodes was $L_{el}=(5\pm1)\cdot10^{-3}$ m, with an area $A_{el}=(1.4\pm0.1)\cdot10^{-4}$ m^2 in contact with liquid. No visible traces of particle adhesion to the ITO were observed during these measurements.

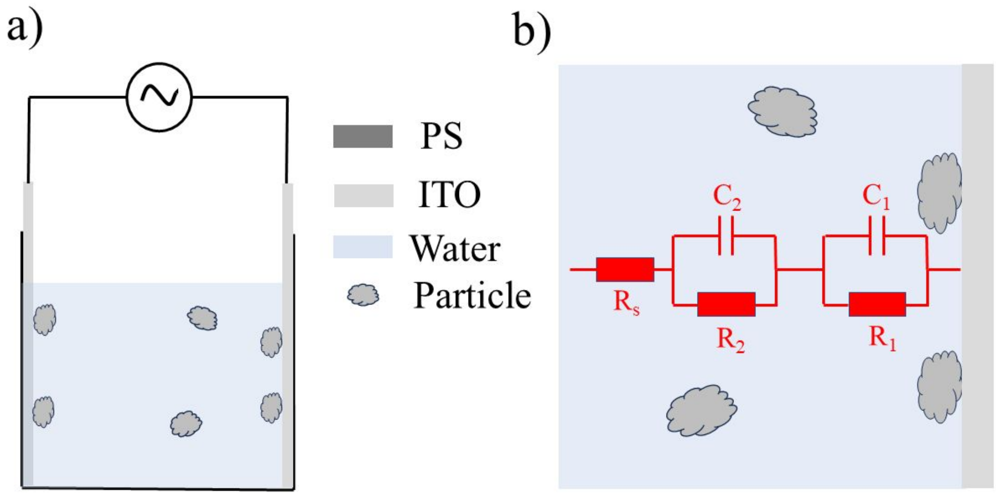

Figure S1. a) The setup used to do impedance spectroscopy on the particle solutions. b) The electrical equivalent circuit used to model the impedance spectra. The symbols are explained in the text.

Bode plots of the impedance spectra are shown for four different concentrations of TiO_2 in water in fig. S2, where fig. S2 a) shows the modulus of the impedance, whereas SI2 b) shows the phase. For

comparison, the spectra for pure water are also shown, and has the largest impedance modulus at f=100 Hz where the blue arrow starts.

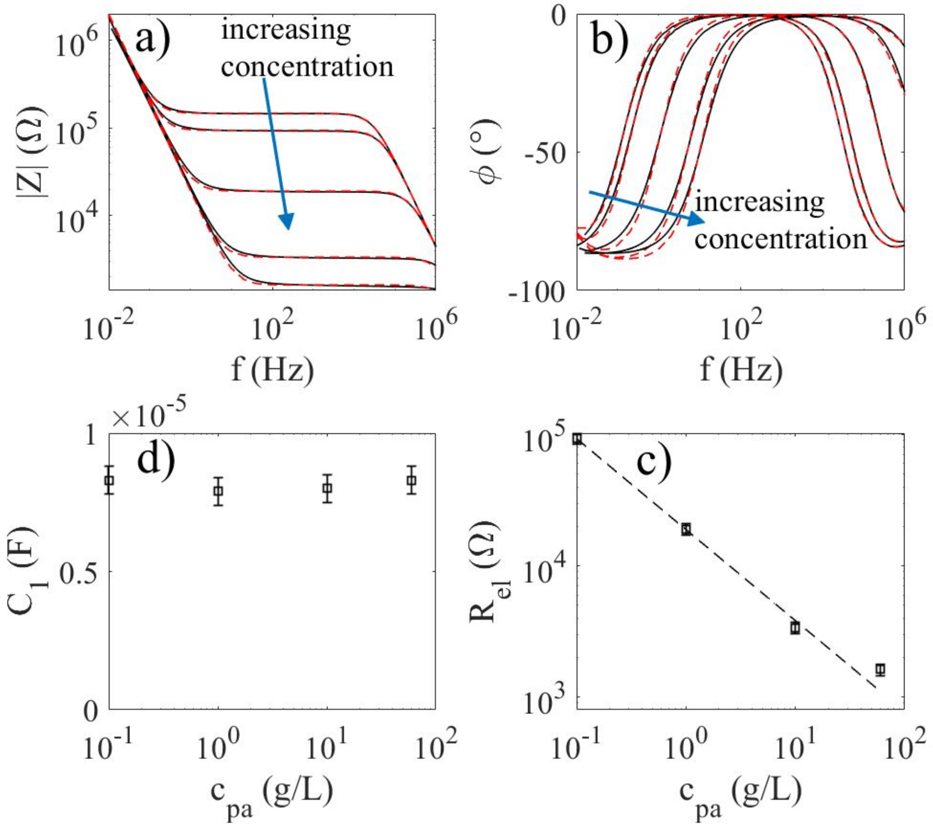

Figure S2. Bode plots with amplitude (a) and phase (b) for four different TiO_2 particle concentrations. The dashed lines are fits of eq. (SI1) to the experimental data, with the electrical equivalent circuit parameters displayed in table I. The extracted electrolyte resistance R_{el} versus particle concentration is shown as squares in c), where the dashed line is a fit of the function $f(c_{pa})=1.9\cdot10^4 c_{pa}^{-0.7}$ to the experimental data. In d), the capacitance C_1 is extracted for different particle concentrations.

The simple equivalent circuit of fig. S1 b) was used to extract information about the conductivity of the particle solutions as well as the capacitance formed by the electrical double at the ITO-water interface. Here, R_s and R_2 represent the contact resistance and the electrolyte, while R_1 is the parallel charge transfer resistance short-circuiting the electrical double layer. The capacitance C_1 is expected to be due to the

electrical double layer, while C_2 is that associated with the two ITO electrodes and could in principle for pure water neglecting electrode edge effects be estimated to be $C_2 \approx \varepsilon_r \varepsilon_0 A_{el}/L_{el} \approx 2 \cdot 10^{-11}$ F, where ε_0 is the permittivity of vacuum while ε_r is the relative permittivity of water. The total impedance is

$$Z = R_s + \frac{R_1}{1+j\omega\tau_1} + \frac{R_2}{1+j\omega\tau_2} \quad , \qquad \text{(SI1)}$$

where $j = \sqrt{-1}$. When $\omega\tau_1$>>1 and $\omega\tau_2$<<1, it is seen that $Z \approx R_{el}=R_s+R_2$, which represents the plateu in fig. S2 a). For smaller frequencies, i.e. $\omega\tau_1$>1 and $\omega\tau_2$<<1, the impedance is $Z=R_s+R_2 + j/\omega C_1$ and the capacitance C_1 is responsible for the slope seen in the left-hand part of fig. S2 a). Using a nonlinear fit of eq. (SI1) to the experimental data in Gamry's Echem Analyst resulted in the values of the equivalent circuit parameters listed in table S1.

c_{pa} (g/L)	C_1 (F)	R_1 (Ω)	C_2 (F)	R_2 (Ω)	R_s (Ω)
0	$8.2 \cdot 10^{-6}$	$1.3 \cdot 10^{7}$	$3.6 \cdot 10^{-11}$	$1.4 \cdot 10^{4}$	$3.8 \cdot 10^{2}$
0.10 (TiO_2)	$8.3 \cdot 10^{-6}$	$1.8 \cdot 10^{7}$	$3.6 \cdot 10^{-11}$	$9.2 \cdot 10^{4}$	$4.4 \cdot 10^{2}$
1.0 (TiO_2)	$7.9 \cdot 10^{-6}$	$1.1 \cdot 10^{7}$	$3.6 \cdot 10^{-11}$	$1.9 \cdot 10^{4}$	$4.5 \cdot 10^{2}$
10 (TiO_2)	$8.0 \cdot 10^{-6}$	$1.1 \cdot 10^{7}$	$5.9 \cdot 10^{-11}$	$2.5 \cdot 10^{3}$	$8.7 \cdot 10^{2}$
60 (TiO_2)	$8.3 \cdot 10^{-6}$	$1.1 \cdot 10^{7}$	$3.0 \cdot 10^{-10}$	$5.1 \cdot 10^{2}$	$1.1 \cdot 10^{3}$
0.10 (C50)	$8.6 \cdot 10^{-6}$	$8.7 \cdot 10^{6}$	$3.7 \cdot 10^{-11}$	$1.2 \cdot 10^{5}$	$2.2 \cdot 10^{2}$
1.0 (C50)	$1.2 \cdot 10^{-5}$	$4.3 \cdot 10^{5}$	$4.0 \cdot 10^{-11}$	$1.7 \cdot 10^{4}$	$3.9 \cdot 10^{2}$
10 (C50)	$1.4 \cdot 10^{-5}$	$4.9 \cdot 10^{3}$	$2.2 \cdot 10^{-6}$	$2.2 \cdot 10^{3}$	$1.7 \cdot 10^{3}$

Table S1: The fitting parameters obtained from fitting eq. (SI1) to the experimental impedance data for carbon (C50) and TiO_2.

In fig. S2 c), the extracted electrolyte resistance R_{el} versus particle concentration is shown as squares in, and the dashed line is a fit of the function $f(c_{pa})=1.9\cdot10^4 c_{pa}^{-0.7}$ to the experimental data. The electrolyte resistance is $R_{el}=L_{el}/(\sigma A_{el})$, where L_{el} is the distance between the electrodes, A_{el} is their area and the electrolyte conductivity is given by σ. This means that is not directly proportional to the particle concentration. On the other hand, the electrical double layer capacitance is nearly independent of the particle concentration as shown in fig. S2 d) and table S1. This may suggest that the thickness of the inner charge layer near the electrode does not change as the particle concentration increases.

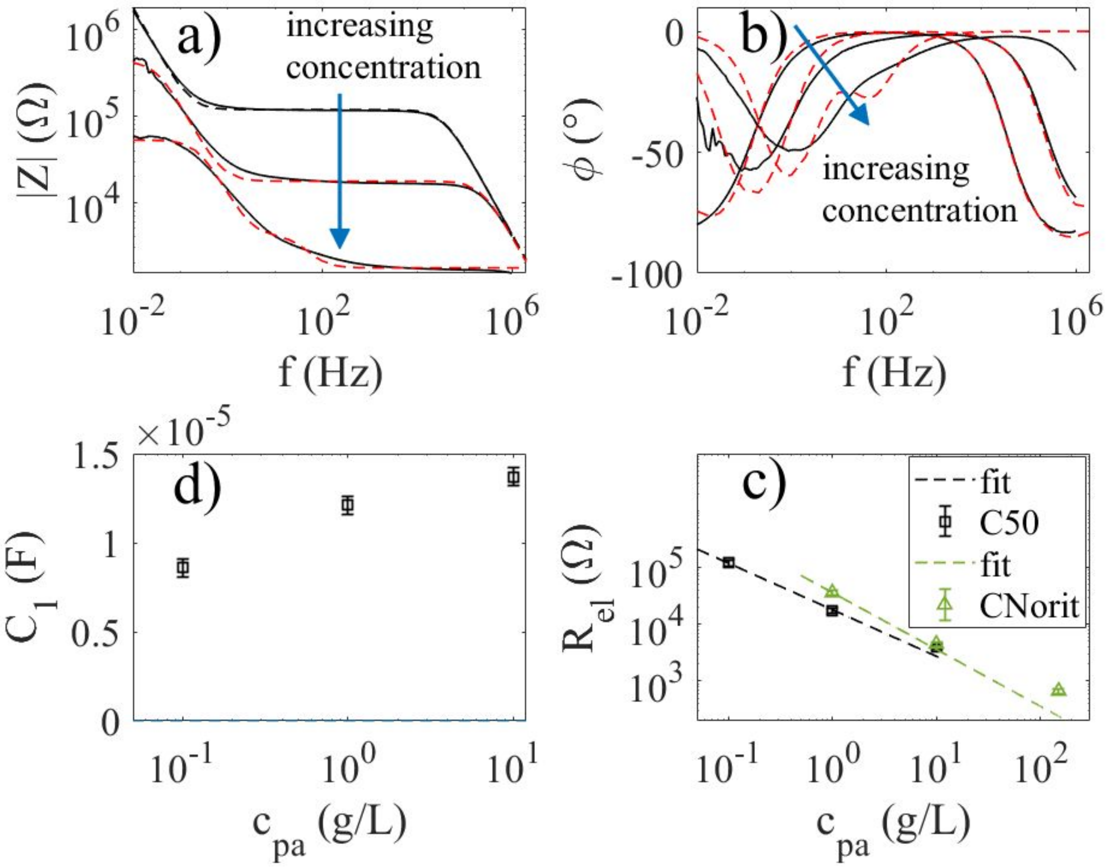

Figure S3. Bode plots with amplitude (a) and phase (b) for three different C50 particle concentrations. The dashed lines are fits of eq. (SI1) to the experimental data, with the electrical equivalent circuit parameters displayed in table S1. The extracted electrolyte resistance R_{el} versus particle concentration is shown as black squares in c). Also plotted in c) is the electrolyte resistance for CNorit for three different particle concentrations (green triangles). The dashed lines are fits to the experimental data. In d), the capacitance C_1 is extracted for different C50 particle concentrations.

Figure S3 shows Bode plots, i.e. the amplitude (a) and phase (b) of three different concentrations of C50 with ITO electrodes. For small concentrations (0.1 g/L or below), the behavior is similar to that of TiO_2, but for larger concentrations there are deviations. The extracted electrolyte resistance R_{el} is shown in fig. S3 c). The black dashed line is a fit of the function $f(c_{pa})=1.8\cdot 10^4 c_{pa}^{-0.8}$. Also here there are some deviations from an ideally expected inverse linear relationship between the particle concentration and the resistance. The C_1 is seen to increase with concentration as seen in fig. S3 d). This could either be due to a thickness-dependence of the diffuse electrical double layer, or that the carbon particles interact with the ITO electrodes more strongly than seen for TiO_2. Here, we are mostly interested in the electrolyte resistance of C50 and CNorit, since only these two particle types give rise to an increase in charge transfer with concentration, suggesting that ion concentration plays a role. We have therefore plotted R_{el} for CNorit as green triangles in fig. S3 c). Also for these particles R_{el} decreases, and the conductivity σ increases, with particle concentration c_{pa}. The best inversely linear relationship is shown as a green, dashed line with $f(c_{pa})=3.6\cdot 10^4 c_{pa}^{-1}$.

From the data for R_{el} an attempt is made to extract the relationship between the particle concentration and the resulting ion concentration. Assume monovalent ions of mobility $\mu=e/(6\pi\eta a)$, where e is the electronic

charge, η is the water viscosity and a is the hydrated ion radius (typically between $3\cdot10^{-10}$ m and $4\cdot10^{-10}$ m for most relevant ions). The electrolyte conductivity is given by $\sigma=1000cF\mu$, where the Faraday constant is F=96485 C/mol and the factor 1000 is included since c is given in mol/liter (not mol/m^3). The electrolyte resistance is $R_{el}=L_{el}/(\sigma A_{el})$, where L_{el} is the distance between the electrodes and A_{el} is their area. This results in the relationship $R_{el}= G_{th}/c$, where $G_{th} =(6\pi\eta aL_{el}/1000A_{el}eF) \approx 18$ Ωmole/L if $\eta=1\cdot10^{-3}$ Pas, $a=3\cdot10^{-10}$ m, $L_{el}=5\cdot10^{-3}$ m, $A_{el}=1\cdot10^{-4}$ m^2 and $e=1.6\cdot10^{-19}$ C. If we assume that the experimental data can be approximated by an inverse linear relationship, a linear fit can be made to the experimental data for C50 in fig. S3 c) with $R_{el} \approx G_e/c_{pa}$ where $G_e = 1.8\cdot10^4$ Ωg/L. With these assumptions one obtains a linear relationship $c=Gc_{pa}$, where $G \approx G_{th}/G_e \approx 10^{-3}$ mol/g. For CNorit, a similar estimate can be made, but now $R_{el} \approx G_e/c_{pa}$ with $G_e = 3.6\cdot10^4$ Ωg/L. Thus, the data for CNorit can be used to estimate a linear relationship $c=Gc_{pa}$, with $G \approx G_{th}/G_e \approx 5\cdot10^{-4}$ mol/g. These simple estimates suggests that 1 mmol ions for C50 and 0.5 mmol for CNorit are released per gram particles. It should be pointed out that linear fits do not appear to give the best approximation to the experimental data. The data in fig. S3 c) suggest that R_{el} is at low particle concentrations slightly higher for CNorit than for C50, which could justify a slightly higher G for C50 at the concentrations. However, for higher concentrations the higher G is not justified. Moreover, it should be emphasized that these estimates are rather uncertain, since the details of the ionic species has not been quantified.

In the case of TiO_2, the data for R_{el} from table 1 allows one to estimate $G \approx 10^{-3}$ mol/g for concentrations below 1 g/L. The highest concentration used to probe charge transfer and particle deposition is 0.4 g/L, which would then suggest a concentration of the order of 0.4 mM.
